# Possible applications of salvianolic acid B against different cancers

**DOI:** 10.37349/etat.2020.00014

**Published:** 2020-08-31

**Authors:** Iram Shahzadi, Zain Ali, Sidra Bukhari, Acharan S Narula, Bushra Mirza, Reza Mohammadinejad

**Affiliations:** 1 Plant Molecular Biology Lab, Institute of Biological Sciences, Department of Biochemistry, Quaid i Azam University, Islamabad 45320, Pakistan; 2 Molecular Cancer Therapeutics Lab, Institute of Biological Sciences, Department of Biochemistry, Quaid i Azam University, Islamabad 45320, Pakistan; 3 Narula Research, Chapel Hill, NC 27516, USA; 4 Neuroscience Research Center, Institute of Neuropharmacology, Kerman University of Medical Sciences, Kerman 7619813159, Iran; National University of Singapore, Singapore

**Keywords:** Salvianolic acid B, polyphenols, apoptosis, molecular targets, cancer

## Abstract

Cancer is the second death causing disease worldwide after cardiovascular abnormalities. The difficulty in treating tumor cells with more precise targeted interventions and recurrence of cancer after treatment may pose great difficulty in developing sustainable therapeutic regimens. These limitations have prompted the need to explore several compounds with ability to cease tumor growth while at the same time induce apoptosis of tumor cells. Several studies have emphasized the use of natural compounds as antitumor agents due to their high efficacy against cancer cells and low toxicity in normal cells. Salvianolic acid B (SAB), a naturally occurring phenolic compound extracted from the radix of Chinese herb *Salvia miltiorrhiza* can induce apoptosis in different types of tumor cells. It can be used to treat cardiovascular and neurodegenerative disorders, hepatic fibrosis, and cancers. Several studies have shown that SAB can mitigate tumorigenesis by modulating MAPK, PI3K/AKT, and NF-ĸB signaling pathways. It also sensitizes the tumor cells to different anti-cancer agents by reversing the multi-drug resistance mechanisms found in tumor cells. This review summarizes the studies showing antitumor potential of SAB in different types of cancer cell lines, animal models and highlights the possible mechanisms through which SAB can induce apoptosis, inhibit growth and metastasis in tumor cells. Moreover, the possible role of nano-technological approaches to induce targeted delivery of SAB to eradicate tumor cells has been also discussed.

## Introduction

A report published by World Health Organization (WHO) has documented that cancer is the second highest cause for death around the world and 70% of deaths because of different cancers have been reported in middle- and low-income countries. Cancer rates could increase by 50% to 15 million new cases a year by the year 2020 [1–3]. Cancer is mainly caused by various mutations in the genome, which can cause deregulation of diverse molecular signaling cascades [4–6]. The presence of constant growth signals, unresponsiveness to antigrowth signals, apoptosis resistant, heightened angiogenesis, tissue invasion in addition to metastasis, enhanced replicative potential, as well as genome variabilities are the major signs of cancerous cell growth [7]. Numerous therapeutic approaches against cancer have been established over the last few decades after acquiring a deeper understanding of several underlying signaling mechanisms that can lead to the enhanced survival and proliferation of neoplastic cells. They include adjuvant and neoadjuvant chemotherapy, targeted therapy, immunotherapy, surgery, and radiotherapy [8–11]. The prevalence of cancer and mortality remains high despite substantial improvements in treatment procedures [12]. This phenomenon can be generally attributed to the limited effects of existing anti-cancer therapies and the expensive cost of the treatment, in addition to substantial adverse reactions [13]. Additionally, modern cytotoxic agents commonly possess life-threatening toxicity [14, 15]. In the past six decades, there have been several cases of removal of pharmaceutical products from the market because of antagonistic drug reactions, with the most prominent adverse event being hepatotoxicity [16]. Additionally, some cancers such as breast cancer may often reappear after remaining inactive for a long time even after successful treatment [17], indicating that discovery of new and safe treatment methods is still needed.

Mother Nature is a reservoir of a significant number of plant-based natural products, which possess significant anti-cancer potential [18]. Several plant-based molecules can act as a chemosensitizer as well as overcome chemoresistance in different types of cancer [19–23]. In addition, 40% of the medicines approved by the FDA available in the market have been derived from products obtained from plants, 74% of which are anticancer drugs [24, 25]. The research in this field has emphasized on the utilization of undiscovered reservoirs of phytochemicals such as alkaloids, glycosides, terpenoids, phenolics, and saponins, in order to avoid the harmful side effects of medications used in chemotherapy, to prolong recovery time as well as to boost the quality of life in cancer patients [5, 19, 26, 27]. *Salvia miltiorrhiza* (*S. miltiorrhiza*, Danshen) belongs to the family Labiatae, is a well-known traditional Chinese herb. In addition, due to its excellent medicinal properties, it has been in use for thousands of years to treat many diseases and is regarded as “Super-grade drug” in Pen-Ts’ao of Shen-Nung [28]. Traditionally, Danshen has been extensively used to treat cardiovascular diseases, mental agitation, memory weakness, insomnia, cancer, and liver fibrosis [29–31]. It also delays the development of atherosclerosis [32]. Its role as an anti-hypertensive and anti-platelet aggregation agent also may result in the prevention of cerebral infarction [33]. Danshen may have a role in the elevated expression of certain antioxidant enzymes [34].

Pharmacologically, Danshen has two categories of compounds, i = lipophilic diterpenoid tanshinones, ii = water-soluble phenolic acids [35]. Tanshinones are important antioxidants, anti-cardiovascular, anti-inflammatory, and antitumor agents and are the main ingredient in *S. miltiorrhiza* [36]. The water-soluble phenolic acids possess various bioactivities including those of antioxidant, anticoagulant, anti-thrombotic, antitumor, and anti-HIV [37]. Among the water-soluble phenolic acids, salvianolic acid B (SAB) is the chief component as per official Chinese Pharmacopoeia. Many studies have reported the promising antitumor, neuroprotective and cardio-protective properties of SAB in different model systems [38–40]. This review briefly highlights the important cellular pathways involved in the antitumoral actions of SAB in different cancer cell lines and in animal models and strategies to utilize its potential for cancer therapy.

### SAB chemistry

**Figure 1.**
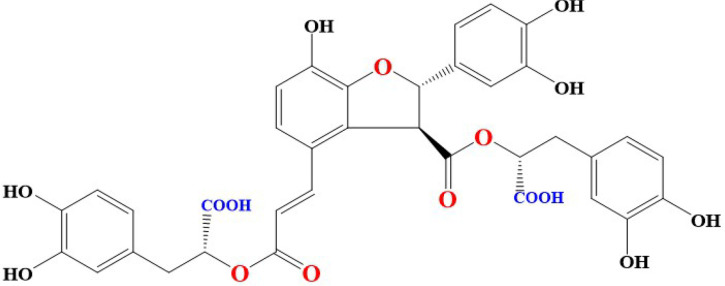
Chemical structure of salvianolic acid B

The basic chemical structure of different derivatives of SAB contain [(*R*)-3-(3, 4-dihydroxyphenyl)-2-hydroxypropanoic acid] and it is also known as lithospermic acid B [41]. Molecular formula of SAB is C_36_H_30_O_16_ and its molecular weight is 718.6138 g/mol. SAB is yellowish and an amorphous compound formed by three Danshensu (Salvianic acid A) molecules and one molecule of caffeic acid. Interestingly, phenolic groups in different compounds are responsible for inhibition of tumor invasion, induction of apoptosis, reversal of drug resistance, modulation of immune response to tumor cells, inhibition of metastasis of tumor cells, and reducing abnormal proliferation [42]. Blocking of lipid peroxidation can be executed by release of hydrogen from activated phenolic hydroxyl groups (9 in number) [43]. Interestingly, antioxidant activities demonstrated by SAB and Danshensu have been attributed to the functional phenolic groups present in their structures [44, 45]. Chemical structure of SAB were shown in Figure 1.

### Biosynthesis of SAB

**Figure 2.**
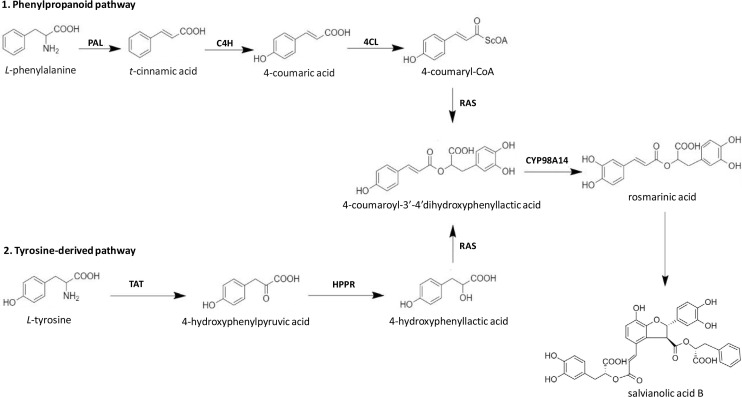
Biosynthetic pathways of SAB in *S. miltiorrhiza*. TAT: tyrosine amino transferase; HPPR: 4-hydroxyphenylpyruvate reductase; PAL: phenylalanine ammonia-lyase; C4H: cinnamic acid 4-hydroxylase; 4CL: 4-coumarate CoA ligase; RAS: rosmarinic acid synthase; CYP98A14: cytochrome P450-dependent monooxygenase

Among different Salvianolic acids, SAB and Rosmarinic acid (RA) can be synthesized primarily through tyrosine derived phenolic acid and phenylpropanoid biosynthetic pathways (Figure 2) [46]. Biosynthesis of RA is initiated when *L*-phenylalanine and *L*-tyrosine are converted to two different intermediates, i.e. 4-Coumaroyl-CoA and 4-hydroxyphenyllactic acid respectively by two parallel but independent pathways. Several subsequent biochemical reactions initiated by covalently joining these two intermediates can form RA at the end. SAB is thought to be derived from RA (Figure 2). However, the detailed mechanism of SAB synthesis has not been studied until date [47]. The extent/quantification of biosynthesis of SAB is dependent on the yield recovered during its extraction process. In general, using conventional reflux-based extraction methods for extraction from roots requires high temperature for a longer time, which may contribute to a lower yield of SAB mainly due to its hydrolysis into tanshinol [48]. However, a higher extraction yield can be achieved over a shorter time and lower temperature when an ultrasound-assisted extraction method is used. Using the ultrasound-assisted extraction method, the yield of salvianolic acid B was 33.93 mg/g in *S. miltiorrhiza* roots higher than those with a conventional refluxing method (28.76 mg/g) [49].

## Various molecular targets affected by SAB

**Figure 3.**
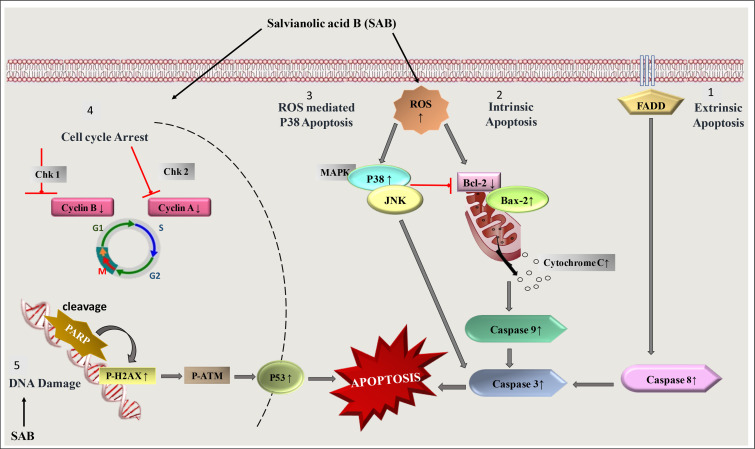
A diagram showing the induction of apoptosis and cell cycle arrest induced by SAB treatment. FADD: FAS-associated death domain protein; JNK: c-Jun N-terminal kinase; MAPK: mitogen-activated protein kinase; Chk: Checkpoints; ↑ increase; ↓ decrease

As cancer is a disease in which several signaling molecules may be deregulated causing the cells to multiply, invade, or metastasize [50]. SAB has been identified as a potential antitumor compound and has been observed to target multiple steps in the apoptotic pathway [51] (Figure 3). The studies on the mechanistic action of SAB showed up-regulation of caspase-9, pro-apoptotic proteins, i.e. B-cell lymphoma 2 (BCL-2)-associated X protein (BAX) and BAK, enhanced caspase-3 level with poly ADP-ribose polymerase (PARP) cleavage and down-regulation of anti-apoptotic proteins such as Bcl-2, which can promote apoptosis [52–55]. In SKOV3 ovarian cancer cells, SAB induced apoptosis by the activation of caspase-3 in a dose-dependent manner [56]. It was also found to cause cell cycle arrest at M/G2 phase by inhibiting the expression of cyclin E and cyclin D [57, 58].

In addition, among several signal transduction pathways, phosphatidylinositol 3-kinase (PI3K)/protein kinase B (AKT) has been found to be deregulated in most types of tumor cells. PI3K/AKT, a serine-threonine kinase protein binds to phosphatidylinositol triphosphate (PIP3) on the surface of a cell and its activity can be modulated by phosphoinositide dependent kinase-1 (PDK-1) after its binding to PIP3. Activated AKT can phosphorylate downstream target proteins, including forkhead box O (FOXO1), glycogen synthase kinase-3 (GSK3β), and mTOR, resulting in cancer cell survival, cell-cycle progression, ribosome biogenesis, and/or protein synthesis [59]. AKT and mTOR are the key proteins involved in regulation of apoptotic and autophagy pathways in different tumor cells. Therefore, it represents a potential antitumor target in therapeutic studies [60–62]. SAB has been found to induce apoptosis and autophagy by inhibiting AKT/PI3K mediated activation of mTOR pathway in colorectal [63] and hepatocellular carcinoma (HCC) cells [60]. Autophagy is an important physiological process, which has been reported to be involved in the maintenance of cellular homeostasis by degrading old proteins and damaged cellular organelles [64]. Autophagy plays a dual role in cancer. It may be involved in tumor suppression as well as in increased proliferation. Apoptosis and autophagy could be induced by the same stimulus, but the interaction between them was still unclear [65]. Most studies have shown effects of SAB on PI3K/AKT signaling pathway resulting in down-regulation of m-TOR. There is need to explore further molecular targets modulated by SAB within PI3K/AKT pathway, i.e. FOXOs and GSK3β as they are also involved in the process of cell survival and cell cycle progression in tumor cells [66].

**Figure 4.**
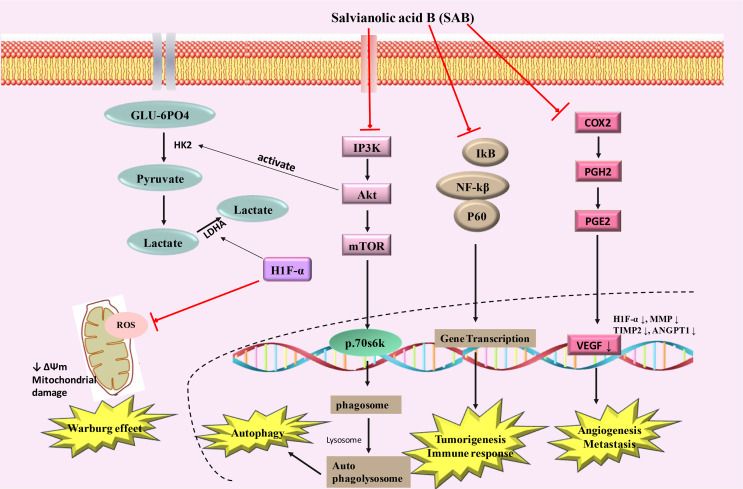
Schematic diagram showing SAB inducing mechanism of action in cancer. PGH2: prostaglandin H2; PGE2: prostaglandin E2; VEGF: vascular endothelial growth factor; MMP: matrix metalloproteinases; IP3K: indole phosphatide 3 kinase; GLU-6PO4: glucose 6 phosphate; ↑ increase; ↓ decrease

Tumor cells exhibit elevated activation of constitutive nuclear factor kappa light chain enhancer of activated B cells (NF-κB), which may lead to increased cell growth, reduced apoptosis, metastasis of tumor cells, angiogenesis and alter cellular metabolism by regulating the expression of many genes such as cyclooxygenase-2 (Cox-2) [67–69]. NF-κB activation can cause tumor cells to become resistant to apoptosis and it can facilitate increased proliferation and metastasis [70–72]. NF-κB has been considered as an important target for modulation by different therapeutic approaches in many tumor types [73, 74]. NF-κB and MDM-2 (mouse double minute 2 homolog) were found to be down-regulated in SAB treated JHU-013 head and neck cancer cells in a dose-dependent sequential inhibition of LPS-stimulated Cox-2 and PGE-2 [57, 58]. The mechanism(s) by which SAB can exert its inhibitory effects on NF-κB pathway is still unclear and requires detailed studies. In 7,12-dimethylbenzanthracene (DMBA) treated hamster model, modifications of key metabolic pathways, including elevated glutaminolysis and glycolysis, and decreased cholesterol and myo-inositol metabolism were observed, which were attenuated by SAB exposure. SAB also inhibited important regulators of cellular proliferation and tumorigenesis, i.e. hypoxia induced factor (HIF)-1α, matrix metalloproteinase (MMP)-9, and tumor necrosis factor (TNF)α [75, 76]. The action of SAB in modulating Warburg effect and affecting tumor growth were summarized in Figure 4.

A brief overview of anti-cancer potential of SAB in different tumor malignancies, i.e. breast cancer, HCC, leukemia, colorectal cancer, head and neck squamous cell carcinoma (HNSCC), glioma cells, cervical cancer, ovarian cancer, and retinoblastoma has been shown in tables 1 and 2. The detailed pharmacological impact of SAB against different cancers has been discussed briefly below:

**Table 1.** Selected anticancer effects of SAB on tumor cell lines

**Type of cancer**	**Model/cell line**	**Morphological effects**	**Mechanisms of action**	**References**
Oral cancer	CAL27, SCC4, Leuk1	Apoptosis, inhibits cell growth, anti-angiogenesis	↓HIF-1α, ↓TNFα, ↓MMP9, ↓Tenascin-C, ↓Osteopontin, ↓TGFβ, ↓Cox-2, ↓HGF, ↓MMP2, ↑THBS2	[75, 76]
	CAL27, HN4, and Leuk1	Apoptosis, inhibits cell growth, modulates Warburg effect	↓MMP, ↓PI3K/Akt/HIF-α	[123]
Leukemia	HL-60	Apoptosis, inhibits cell growth	-	[125]
Cervical cancer	Hela cells SAB + ATO	Apoptosis, inhibits cell growth	↓pro-caspase 3, ↑PARP cleavage	[96]
Retinoblastoma	HXO-RB44	Apoptosis, cell volume shrinkage, chromatics agglutination, inhibits cell growth, cell cycle arrest at S phase	↑caspase 3	[117]

↑ increases expression, ↓ decreases expression

**Table 2.** Selected *in vivo* anticancer effects of SAB

**Type of cancer**	**Mouse/mice/hamster model**	**Morphological effects**	**Mechanism(s) of action**	**References**
Breast cancer	Mouse model	Apoptosis induction, reduction of oxidative stress, anti-inflammatory, anti-angiogenesis	↓MMp-8, ↓TNF, ↓COX-2, ↓p53, ↑caspase 3	[83]
	Mouse model	Increased cell apoptosis, inhibition of growth	↓PCNA, ↓Survivin, ↓BCL-XL	[84]
Colon cancer	LoVo cells HCT-116 cells nude mice	Inhibits tumor growth, inhibits tumor invasion, multidrug resistance	↓CD44, ↓CD133, ↓SOX-2, ↓ABCG2	[108]
	BALB/c nude mice injected with HCT116 cells	Pro-death autophagy	↑Atg5 expression, ┴AKT/mTOR signaling pathway, ↓p70S6K	[63]
Glioma	U87 xenograft nude mice	Tumor volume reduced, weight reduced, increases ROS	↑p38, ↑p53	[111]
Oral cancer	Hamster	Antiproliferative, inhibition of angiogenesis	↓HIF-1α, ↓VEGF	[122, 123]
Head and neck cancer	JHU-013 xenograft mouse	Apoptosis, anti-proliferative, angiogenesis	↓COX-2	[57, 58]

↑ increase expression, ↓ decrease expression, ┴ inhibit

### Breast cancer

Breast cancer remains a leading cause of cancer related deaths in females worldwide [77–80]. Chemotherapy is the most common way to treat breast tumors despite some limitations including high toxicity, normal cell death, and increase in drug resistance [81, 82]. The proliferation of hormone receptor-positive breast cancer cell line (MCF-7) was decreased by SAB *in vitro*. It also attenuated the tumor volume and increases the survival of Ehrlich solid carcinoma cell line (ESC) injected mice. Furthermore, in a preclinical study, SAB decreased the plasma level of glutathione (GSH) and malondialdehyde in mice. In ESC injected mice, it decreased the expression of MMP-8, TNFα, Cox-2, and level of cyclin D1 in combination with cisplatin. It also increased the expression level of p53 and caspase-3 [83]. Triple-negative breast cancer (TNBC) is an aggressive subtype of breast cancer with limited treatment options [82]. Moreover, an *in-vitro* study showed a significant reduction in proliferation and decreased expression of cyclin B1 expression in hormone receptor-positive MCF-7 and triple-negative MDA-MB-231 breast cancer cell lines by SAB. In mouse model, the inhibition of growth, increased apoptosis, and decreased expression of proliferating cell nuclear antigen (PCNA) was observed in MDA-MB-231 tumor xenograft mouse model. SAB also caused an enhanced accumulation of ceramide and inhibited the expression of survivin and Bcl-xL [84]. These effects showed that SAB could be an effective therapeutic compound against breast cancer.

### HCC

HCC is the carcinoma of liver, which ranks third at causing cancer related deaths and is the fifth most common cancer of the world [85–87]. Mostly hepatitis B and C virus are associated with the HCC development in the patients [88]. Different chemotherapeutic drugs as well as targeted therapies are available for HCC treatment but now due to their numerous side effects, natural drugs are also being used [6, 25, 89–94]. In an earlier study, it was observed that SAB can inhibit the cell proliferation at a higher dose, downregulate the expression of cytochromes CYP1A2 and CYP3A4 and upregulate the expression of GSH S-transferase (GST) in HepG2 human hepatoma cell lines [95]. The combination of SAB with arsenic trioxide (ATO) enhanced the cytotoxicity of ATO as well as induced apoptosis in HepG2 cell lines with decreased expression of procaspase-3 and increased expression of cleaved PARP that is an apoptotic marker [96].

SAB has also shown the potential to inhibit the growth of SK-Hep-1 and Bel-7404 HCC cell lines, induce autophagy, and promote apoptosis through activating mitochondrial pathway leading to cancer cell death. This can be achieved by inhibition of AKT/mTOR signaling pathway *in-vitro* in HCC lines. SAB downregulated the mTOR levels, phosphorylated AKT along with its downstream effector p-4EBP1 and p70S6K proteins, thus inhibiting cellular growth, proliferation, and metabolism. Suppression of autophagy by pharmacological inhibitors (3-MA and CQ) or Beclin-1 siRNA decreased SAB-induced apoptosis, thus revealing the role of autophagy in promoting apoptosis [60].

### HNSCC

Head and neck cancer rank among the top ten cancers [97–99]. This cancer is mainly associated with tobacco exposure [100] and a number of inflammatory pathways are involved in the development of HNSCC [101, 102]. Different studies have indicated that SAB can exhibit chemo-preventive effects against HNSCC [103]. SAB caused a decrease in the expression of Cox-2 and induced apoptosis in a variety of head and neck carcinoma [58]. SAB attenuated the tumor growth in JHU-013 xenograft mice and decreased the expression of Cox-2 substantially with apoptosis induction [57, 58]. It also inhibited proliferation in four HNSCC cell lines (JHU-06, JHU-011, JHU-013, and JHU-022) [103].

### Colorectal cancer

Colorectal cancer is the third most death causing cancer worldwide [104, 105]. In colorectal cancer patients, 5-year survival rate generally after diagnosis is around 50–55% [106]. Chemotherapy for colorectal cancer is the ideal choice of intervention but drug resistance often hampers the success of therapy. Thus, there is an urgent need to address drug resistance in colorectal cancer. SAB reduced cell proliferation and increased apoptosis in colorectal cancer cells (HCT-8/VCR) at different concentrations. SAB showed IC_20_ (concentration needed to kill cells by 20% as compared to untreated control) of 20.79 ± 4.76 µg/mL and IC_50_ (concentration needed to kill cells by 50% as compared to untreated control) of 114.79 ± 10.94 µg/mL against HCT-8/VCR cells. At non-toxic concentration, SAB enhanced the effects of VCR, CDDP, Taxol, and 5-fluorouracil (5-FU) by inhibiting drug resistance in colorectal cancer cells. SAB also reversed the multi-drug resistance (MDR) of colorectal cancer cells by causing a down-regulation of P-gp protein, which subsequently enhanced the sensitivity of colorectal cells to these drugs and increased expression of pro-apoptotic mitochondrial protein Bax while causing a down-regulation of anti-apoptotic Bcl-2. SAB enhanced ROS production that leads to reduction in mitochondrial membrane potential and increased cell death [107]. *In-vivo* studies using nude mice injected with colorectal cancer cells (LoVo & HCT-116) showed reversal of drug resistance, reduced tumor cell invasion, and increased apoptosis. Tumor invasion markers such as CD44, CD133, ABCG-2 and *Sox-2* were down-regulated. In addition, 5-FU and L-OHP showed greater efficacy, reduced tumor growth, and increased apoptosis when injected in combination with SAB as compared to 5-FU and L-OHP alone. These findings suggested potential of SAB to attenuate drug resistance in colorectal cells [108].

Moreover, SAB induced substantial autophagy in HCT116 and HT29 colorectal cells and *in-vivo* in HCT116 injected BALB/c nude mice. The expression of caspase-3, caspase-9, and PARP was elevated after SAB exposure. It also induced autophagy by inhibiting AKT/mTOR signaling pathways. Transfection of AKT plasmid in colorectal cells reduced SAB induced autophagy while repression of AKT signaling pathway by LY294002 (PI3K inhibitor) increased SAB mediated autophagy [63].

### Glioma

Glioma is the leading cause of brain-related tumor’s death worldwide and it can affect the central nervous system [109]. The rate of survival is generally less than 5% and the average life expectancy of individuals diagnosed with glioblastoma, an aggressive subtype of Glioma is 12–14 months [110]. Treatment of human primary glioblastoma cell line (U87) with 50 µmol/L SAB inhibited growth and increased apoptosis in a dose-dependent manner. SAB treated U87 glioma xenograft nude mice showed reduced tumor volume and weight. The apoptosis induction and reduced growth in glioma cells was primarily mediated through p38MAPK and p53 activated ROS pathways [111].

Radiation therapy is the standard mode of treatment in glioma patients. There is an increased resistance to radiation by glioma cells, which may result in the failure of therapy. At 0.5 µmol/L concentration, SAB did not affect the viability of glioma cells but increased the efficacy of radiotherapy in U87 (human primary glioblastoma cell line). SAB did not have any effect on the sensitivity of temozolomide (TMZ) at this concentration. SAB increased the antitumor potential of radiotherapy by increasing mitochondrial fission and activating mitochondrial fission proteins. Overall, SAB rendered radiotherapy more effective in glioma cells by increased mitochondrial fission through Fis-1 mediated mitochondrial fission [112].

### Cervical cancer

Cervical cancer is the fourth most prevalent type of cancer in women [113]. Combinatorial administration of SAB with ATO drug showed enhanced antitumor activity against cervical cancer cells (HeLa) and antitumor effect was found to be apoptosis dependent. As compared to control cells, ATO and SAB treated HeLa cells displayed enhanced caspase-3 mediated PARP cleavage after 48 h of treatment. This indicated that SAB may have an important role as an antitumor drug in cervical cancer [96].

### Ovarian cancer

Ovarian cancer is the fifth most prevalent cancer among women worldwide [114]. Chemotherapy and surgery are the most common type of interventions used to curb the deleterious effects of ovarian cancer. Ovarian tumor cells are prone to develop drug resistance over time and this leads to recurrence of ovarian cancer after treatment [115]. There is an imminent need to explore candidate antitumor compounds, which reduce the growth of ovarian cancer cells with minimal effects on normal cells. SAB showed antitumor potential against ovarian cancer cells (SKOV3) with an IC_50_ value of 45.6 µmol/L. The rate of apoptosis was increased at higher concentration of SAB. In addition, it caused blockage of cell cycle at M phase and G-2 phases of cell cycle and a significant increase in the expression of caspase-3 [56].

### Retinoblastoma

Retinoblastoma is a cancer of children and it contributes 4% of total pediatric cancer prevalence. Its incidence is around 1 out of 18, 000 births [116]. SAB has shown potential as an antitumor drug against retinoblastoma cells (HXO-RB44). It inhibited the growth of HXO-RB44 cells in a time and dose-dependent manner. HXO-RB44 cells showed significant apoptosis and other morphological changes, i.e. shrinkage of cell volume, vacuoles formation, and chromatic agglutination after treatment with 0.7 mg/mL dose of SAB at 24, 48 and 72 h of administration. SAB induced apoptosis and blocked the cell cycle at S-phase so that tumor cells cannot enter G-2 phase and the expression of caspase-3 was significantly high after 48 h of treatment in HXO-RB44 cells. Overall, SAB induced apoptosis, blocked cell cycle and inhibited the proliferation of retinoblastoma cells in concentration and time-dependent manner [117].

### Oral cancer

The number of cases of oral cancer is increasing day by day with decreased survival rate and increased mortality rate [118, 119]. Oral squamous cell carcinoma (OSCC) is most common among carcinoma of oral cavity and becoming an important health care problem [120]. Conventional treatment generally includes surgery, chemotherapy, and radiation therapy. All of these therapies are showing fewer positive responses therefore need for natural and more efficient drugs is necessary [121]. The effect of SAB was analyzed on OSCC cell lines and it caused an inhibition of growth of OSCC cell lines. SAB decreased the proliferation of squamous cell carcinoma (SCC) in DMBA induced oral cancer in Hamsters. Inhibition of angiogenesis, HIF-1α and VEGF protein expression was also observed upon SAB exposure through immunohistochemistry from tissue samples obtained from DMBA induced oral cancer in Hamsters [122]. SAB has also shown promising antitumor effects on oral squamous carcinoma cells (CAL27 and SCC4) by inhibiting their proliferation and inducing apoptosis in a time-dependent manner. However, it did not cause any significant antitumor effects in immortalized oral leukoplakia cells (Leuk1). SAB induced apoptosis and inhibited tumor cells angiogenesis by inhibiting expression of Cox-2, HGF, MMP-2, HIF-1α, TNFα, MMP-9, tenascin, osteopontin as well as transforming growth factor (TGF)-1β and up-regulating the level of THBS-2 [75]. However, additional *in-vivo* studies are required to confirm the effects of SAB on various oncogenic markers, and correlation of these effects with clinical outcome will further expedite the use of SAB as an antitumor drug against oral cancers.

Metabolic modulation by SAB may also mediate its anti-cancer actions against SCC. DMBA induced hamsters showed enhanced glycolysis and glutaminolysis, reduced myoinositol and cholesterol metabolism while SAB treated DMBA induced hamsters showed normal effects as compared to altered metabolic conditions of DMBA induced group. Interleukin 10 (IL-10) mRNA expression was reduced while TIMP-2 and ANGPT-1 expression was increased in SAB treated DMBA injected hamsters [76]. Next-generation sequencing of DMBA injected SAB treated hamsters showed down-regulation of PI3K and HIF-1α signaling pathways. SAB also exhibited inhibitory effects on PI3K-Akt and HIF-1 α pathways in Cal27 and HN4 cell lines. In pre-malignant Leuk1 cells, SAB treatment resulted in loss of mitochondrial membrane potential, reduced colony formation, and enhanced apoptosis. These findings suggest the role of metabolic modulation by SAB in tumor cells by altering PI3K/Akt and HIF-1α signaling pathways [123]. SAB also induced apopstosid and inhubit cell growth by decreasing Cox-2 expression in lumg cancer A549 cell line [124].

## Role of SAB in regulating epithelial-mesenchymal transition (EMT)

For tumor growth and spread, epithelial-mesenchymal transition (EMT) is an important process and enhanced expression of mesenchymal genes (Fibronectin, Vimentin, N-Cadherin), as well as reduced expression of epithelial genes (E-Cadherin), are the most important characteristics of EMT [126, 127]. Metastasis poses the biggest hurdle in effective cancer treatment and accounts for 90% mortality rate caused by different cancers [128–133]. SAB suppressed extracellular matrix modelling and cellular proliferation through inhibition of NF-κB associated activation of MMP-9 and MMP-2 in high glucose induced mesangial cells [134]. In addition, MMP-9 levels were also noted to be significantly downregulated in SAB treated breast cancer cells [83]. Moreover, studies have indicated that SAB may have a role in the inhibition of EMT by modulating the expression levels of different micro-RNAs. Yu et al. [135] showed that administration of SAB reversed liver fibrosis, repressed Hedgehog pathway and EMT by up-regulation of Patched-1, miR-152, and DNA methyl transferase 1 (DNMT1). In addition, miR-106b, miR-93 and miR-25 were significantly downregulated in TGF-β induced EMT [135].

In addition, another study showed a dose-dependent increase in the expression of miR-106b-25 cluster in SAB treated HK-2 human kidney cancer cells. Interestingly, miR-106b can reduce EMT by increasing the expression levels of E-cadherin and lowering expression levels of α-smooth muscle actin (α-SMA) [136]. *In-vivo* studies have also revealed inhibition of TGB-β1-induced EMT in SAB treated HK-2 cells by modulation of TGF-β/Smad signaling pathway [137]. Moreover, a reversal of TGF-β1 induced EMT in KH-2 cervical cancer cells by nano-formulation of SAB (HCA-Chi-Ca-SAB) has been reported [138]. These findings suggest the potential role of Salvianolic acids in the reversal of EMT in cancer. EMT reversal may also result in the regeneration of already disseminated cancerous cells within the body [139]. Further studies on the exploration of mechanistic pathways involved in EMT reversal by SAB will establish its role and usefulness as an anti-EMT drug.

## Pharmacokinetics and bioavailability of SAB

A number of naturally derived biological compounds have deficiency in distribution, rapid metabolism, excretion, and poor absorption that may restrain their bioavailability [140]. Several experiments have been conducted to study the pharmacokinetics behavior of SAB in humans and animals for instance dogs, rabbits, and rats. The pharmacokinetic profile of SAB in rats was studied using two-compartment open models by subsequent oral course of 200 mg/kg. The absolute/standard SAB bioavailability is 0.022%. Several investigations suggested that after oral administration of SAB, 60% of given SAB remains in the gut for at least 180 min, thus contributing to its poor bioavailability in the body [141]. Oral bioavailability in dogs was noted to be 1.1% only, after the administration of SAB doses, i.e. 80 mg/kg orally, and 9 mg/kg [142]. The intravenously injected doses with 3, 6, and 12 mg/kg of magnesium SAB in beagle dogs have also been examined. It was evident from the previous studies that the elimination and distribution of SAB were fast enough [143]. The normal urinary excretion rate of SAB reported from the studies was 0.16%. However, the recovery of SAB from the gastrointestinal (GI) tract was 41.2% and 23.3% respectively when the oral course of 10 and 50 mg/kg doses were applied [143].

The intravenous dose of SAB in humans exhibited 0.29 h half-life of SAB’s elimination with the application of 100 mg/kg dose, although the concentration of SAB gradually and quickly increased in the bile. It attained the highest value within 30 min [144]. It was observed that the concentration of SAB is greater in the bile than in plasma at certain points, which suggested that the hepatobiliary eradication of SAB might imply an active transport [145]. Furthermore, the cumulative absorption concentration of salvianolic acid B was greater in rear jejunum segments than in middle and front segments. SAB has a low oral bioavailability of 4% due to the confined intestinal permeability [146]. Moreover, human epithelial colorectal adenocarcinoma cells (Caco-2) study revealed the lower concentration of SAB in cell membrane permeability. About 5% of SAB bioavailability was noted after oral administration [147]. One hundred mg/kg SAB was administered intravenously, and 500 mg/kg of SAB was administered orally in conscious and freely moving rats. The oral bioavailability of SAB was found to be 2.3% in freely moving rats [148]. Fluorescent poly (ethyl-cyanoacrylate) nanoparticles (300 nm size) were loaded with SAB and it enhanced the bioavailability and sustenance of SAB and allowed the nano formulation to cross blood-brain barrier [149].

## Approaches to ameliorate the bioavailability of SAB

The low bioavailability of SAB can be correlated to the rapid metabolic clearance or its poor absorption in the body. However, two main strategies can be adopted to enhance the SAB absorption, i.e. preparation of fat-soluble complexes and the use of absorption enhancers. The sodium caprateused as co-administration of absorption enhancer can significantly increase the intestinal permeability with the *in vivo* bioavailability of SAB [147]. Borneol is a common Chinese herbal medicine, which can improve the intestinal absorption of SAB in a dose-dependent manner [150, 151]. The production of fat-soluble complexes such as phospholipids may possibly be another suitable preference that can enhance the absorption of SAB in GI tract. This can be attained through the phospholipid complex loaded with the nanoparticle formation. This formulated complex can lead to an increase of 2.9 folds in the relative bioavailability in comparison to the normal salvianolic acid B formulation [152]. Increased bioavailability of SAB has been reported using SAB phospholipid complex loaded pellets without causing any significant increase in C_max_ when compared to SAB alone [153, 154]. This finding supports the utilization of nanoparticle establishment that produces a greater bioavailability than the pellet formation.

The two major metabolites of SAB produced after it was injected intravenously in rats are, i.e. monomethyl-SAB (3-MMS) and Lithospermic acid (LSA). The metabolic pathway for SAB in rats included methylation by means of catechol-*O*-methyltransferase (COMT) in kidney and liver [152]. The blood concentration can greatly be increased in rats by co-administration of an intravenous dose of 50 mg/kg SAB and 0.5 mg/kg ferulic acid [155]. Additionally, the accumulative absorption of SAB in the rats was increased from 3% to 40% in the bile excretion, when used with *L*-DOPA and tolcapone. Likewise, with the single intravenous dose of SAB, the plasma concentration of 3-*O*-methyl dopa (3-OMD) was noted to be decreased. The current information related to CYP enzymes and SAB is debatable. Qui et al. [156] revealed that SAB has no obvious effect on CYP enzyme using human liver microsomes (HLMs).

**Figure 5.**
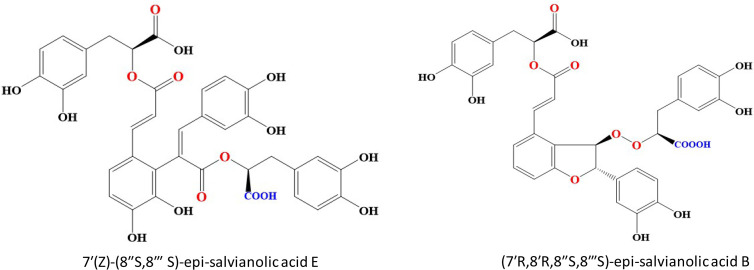
Chemical structures of Salvianolic acid B analogues

In contrast to previous study, SAB was reported to exhibit effectual concentration-dependent inhibitory effect on CYP3A4 activity with IC_50_ values of 1.44 mg/L on HLMs thus demonstrating the role of CYP3A4 substrate [157]. Ferulic acid nano particles (FA-NPs) loaded with SAB displayed significantly stronger antitumor effects via receptor-mediated targeted delivery in MCF-7 and MDA-MB-231 cells [158]. Two novel analogs of SAB were recently isolated from Salvianolic acid injection. 7′(*Z*)-(8″*S*,8‴*S*)-epi-salvianolic acid E (compound 1) is a ring opened product of SAB and (7′*R*,8′*R*,8″*S*,8‴*S*)-epi-salvianolic acid B (compound 2) is a non-enantiomer of SAB (Figure 5). Both these compounds were around 6% of total SAB injection. Compound 1 showed neuroprotective effects comparative to SAB while compound 2 exhibited potent antioxidants effects [159]. Pharmacokinetics study on both SAB analogs and SAB showed that at a dose of 6 mg/kg, compound 1 and compound 2 had a slow elimination rate than SAB. Interestingly, compound 1 and SAB had higher exposure at the same dosage than compound 2. These findings showed the potential therapeutic role of novel analogs of SAB [160].

## Biosafety profile of SAB

Biosafety and toxic profiling of SAB in rats showed significant adverse effects in lungs, liver, kidney, brain, and heart after administration of SAB in combination with ginsenoside Rg1 at a lethal dose (LD_50_) of 1, 747 mg/kg. This dose is 100 times more than the effective dose of SAB [161]. Many clinical trial studies have cleared *S. miltiorrhiza* for the treatment of stroke, heart attack, and many other clinical pathologies [162]. As a non-toxic and bio-safe ingredient in a traditional medicine being used for a century, SAB has primarily emerged as a safe drug for clinical use. However, additional studies are required to explore and validate its safety for human use.

## Conclusion and Future perspectives

The cancer has created havoc for the humanity and its treatment has baffled scientists and researchers for past many decades. The use of natural compounds to treat different types of cancers has a promising future, as these compounds can be effective at doses, which are likely to cause lesser adverse effects. SAB provides an alternative to already existing therapies for different typed of cancer due to its ability to target multiple cellular pathways, i.e. MAPK, PI3K/AKT, and NF-ĸB to induce apoptosis, inhibit invasion and proliferation of tumor cells. It can also sensitize tumor cells to other antitumor agents by reversing the multi-drug resistance mechanisms operational in tumor cells. The limitation of bioavailability of SAB is a major hurdle for its use in treatment, which can be overcome by using nanoparticles-based drug delivery systems to enhance its efficacy and retention in the living systems. Further pre-clinical and clinical studies are essential to cement the idea that SAB can be used effectively in therapy of different types of cancers.

## Abbreviations

5-FU: 5-fluorouracil
AKT: protein kinase B
ATO: arsenic trioxide
BAX: BCL-2-associated X protein
BCL-2: B-cell lymphoma-2
Cox-2: cyclooxygenase-2
DMBA: 7, 12-dimethylbenzanthracene
EMT: epithelial-mesenchymal transition
ESC: Ehrlich solid carcinoma cell line
HCC: hepatocellular carcinoma
HIF: hypoxia induced factor
HNSCC: head and neck squamous cell carcinoma
IC_50_: concentration needed to kill cells by 50% as compared to untreated control
MAPK: mitogen-activated protein kinase
MMP: matrix metalloproteinase
NF-κB: nuclear factor kappa light chain enhancer of activated B cells
OSCC: oral squamous cell carcinoma
PARP: poly ADP-ribose polymerase
PI3K: phosphatidylinositol 3-kinase
*S. miltiorrhiza*: *Salvia miltiorrhiza*
SAB: salvianolic acid B
SCC: squamous cell carcinoma
TGF: transforming growth factor
TNF: tumor necrosis factor
VEGF: vascular endothelial growth factor

## Declarations

### Copyright

© The Author(s) 2020.
